# Post-Translational Modification of ZEB Family Members in Cancer Progression

**DOI:** 10.3390/ijms232315127

**Published:** 2022-12-01

**Authors:** Mi Kyung Park, Ho Lee, Chang Hoon Lee

**Affiliations:** 1 Department of Cancer Biomedical Science, Graduate School of Cancer Science and Policy, National Cancer Center, Goyang-si 10408, Republic of Korea; 2 College of Pharmacy, Dongguk University, Seoul 04620, Republic of Korea

**Keywords:** zinc finger E-box binding homeobox (ZEB), post-translational modifications (PTM), cancer progression

## Abstract

Post-translational modification (PTM), the essential regulatory mechanisms of proteins, play essential roles in physiological and pathological processes. In addition, PTM functions in tumour development and progression. Zinc finger E-box binding homeobox (ZEB) family homeodomain transcription factors, such as ZEB1 and ZEB2, play a pivotal role in tumour progression and metastasis by induction epithelial-mesenchymal transition (EMT), with activation of stem cell traits, immune evasion and epigenetic reprogramming. However, the relationship between ZEB family members’ post-translational modification (PTM) and tumourigenesis remains largely unknown. Therefore, we focussed on the PTM of ZEBs and potential therapeutic approaches in cancer progression. This review provides an overview of the diverse functions of ZEBs in cancer and the mechanisms and therapeutic implications that target ZEB family members’ PTMs.

## 1. Introduction

Cancer-associated mortality represents the second leading cause of death worldwide after cardiovascular disease [1]. Cancer metastasis is the primary cause of cancer mortality, accounting for approximately 90% of tumour-related deaths. The epithelial-mesenchymal transition (EMT) is the tissue repair and developmental process, along with neural crest formation, heart morphogenesis, and mesoderm formation, facilitating gastrulation and secondary palate formation [2,3,4,5]. Moreover, EMT is a vital clue to tumour invasion and metastasis. Zinc finger E-box binding homeobox transcription factors (ZEBs) play a crucial role in the progression and metastasis of various cancers, as EMT-related transcription factors [6,7,8,9,10,11,12,13], in the regulation of DNA damage repair [14] and neuronal differentiation [15].

Furthermore, ZEBs are associated with the degree of malignancy in various types of cancer and the activation of EMT signalling, which are widely believed to contribute to invasion, metastasis, recurrence and therapeutic resistance. ZEBs are also associated with cancer transformation and EMT. Post-translational modification (PTM) is the enzymatic modification of proteins after synthesis [16] and induces proliferation in cancer progression by regulating the cell cycle, cell survival and cellular signalling [17]. In addition, it is found through structural studies and biochemical studies that cofactors present through covalent bonds at the active sites of enzymes also undergo the PTM process [18,19,20]. Therefore, the PTM of proteins plays a regulatory role in cancer initiation and progression. In turn, the ZEBs are regulated by PTM, including phosphorylation, SUMOylation, ubiquitination, acetylation and deacetylation. This review focuses on the specialised roles of various ZEBs’ PTMs on cancer progression.

## 2. ZEB1 and ZEB2 Proteins and Their Physiological Functions

**Figure 1:**
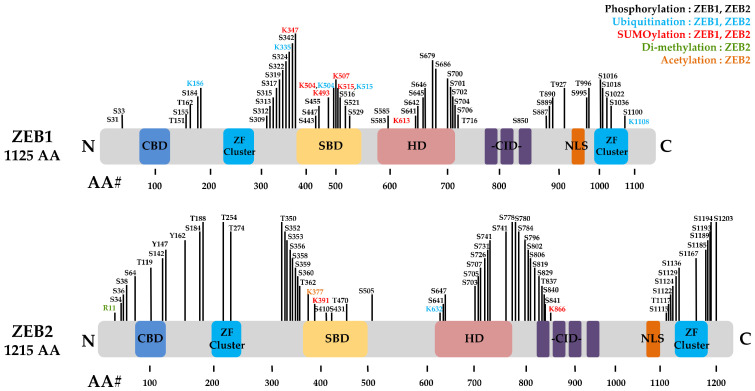
Overviews of ZEB1 and ZEB2 PTMs. It is characterized by the presence of two zinc finger clusters, one at each end (NZF and CZF) and located homeodomain (HD). Other domains are P300-P/CAF interaction domain (CBD), the Smad binding domain (SBD) and the CtBP interaction domain (CID). ZF, zinc finger; NLS, nuclear localization signal. PTM site. Black, Phosphorylation (ZEB1, ZEB2); Sky blue, Ubiquitination (ZEB1, ZEB2); Red, SUMOylation (ZEB1, ZEB2); Green, Di-methylation (ZEB1, ZEB2); Orange, Acetylation (ZEB2).

The zinc finger E-box-binding homeobox 1 (ZEB1) is also known as δEF1, ZFHX1A, MEB1, Nil-2-a, TCF8, AREB6, ZFHEP1 or BZP [21]. The human *ZEB1* gene is located on chromosome 10p11.22 and encodes the 1117 amino acid ZEB1 protein [22]. Zinc finger E-box-binding homeobox 2 (ZEB2) is identified as KIAA0569, SIP1, ZFHX1B and ZFX1B; the human *ZEB2* gene is located on chromosome 2q22.3 and encodes a 1214 amino acid protein [23]. The ZEB proteins consist of a homeodomain (HD) in the middle of the structure and other protein binding domains, including the SMAD interaction domain (SID), which regulates the transforming growth factor beta (TGFβ)-mediated transcription with bone morphogenetic proteins (BMP) signalling, zinc finger domain (ZFD), coactivator binding domain (CBD), CtBP interaction domain (CID) and the p300-CBP-associated factor (P/CAF) binding domain, which control EMT as a trigger of for tumour progression and metastasis (Figure 1) [24,25,26,27,28].

ZEB1 can recruit cosuppressors or coactivators by high-affinity binding of the ZFD to specific DNA binding sites (CACCTG), upregulating or downregulating its target genes [29]. ZEB proteins bind to SMADs. However, while ZEB-1/dEF1 synergises with SMAD proteins to activate transcription, promote osteoblastic differentiation and induce cell growth arrest, ZEB1 is expressed during development in the central nervous system, heart, skeletal muscle and haematopoietic cells; this plays pivotal roles in regulating development, differentiation and maintenance [26,30].

Additionally, ZEB1 is a transcriptional activator, or, has repressor functions in normal regulatory processes and dysregulated progress, such as cancer progression and metastasis. ZEB2 is expressed during the development in the neural tube and crest cells and all parts of the developing forebrain. In addition, it plays a role as a regulator of the TGFβ/BMP signal pathway. When the TGFβ/BMP factor binds to the receptor, the SMAD proteins are translocated to the nucleus, activating the target genes’ transcription. ZEB2 interacts with R-SMADs to induce embryo neutralisation and disrupts the expression of the activin-dependent *Brachyury* gene in *Xenopus* [31,32]. ZEB2 also endures post-transcriptional regulation by several micro-RNAs (miRNAs), such as postnatal brain miRNA (miR9) [33].

## 3. ZEB1 and ZEB2 in Cancer Progression

**Figure 2:**
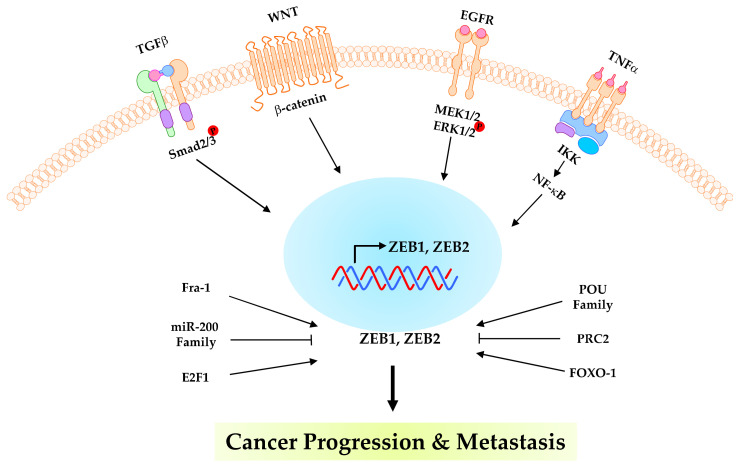
Mechanisms of ZEB family in cancer progression and metastasis. EGFR, WNT, tumor necrosis factor-a (TNFa), transforming growth factor beta (TGF-b), Fos-related antigen 1 (Fra-1), miR-200 family, POU family, PRC2 and FOXO-1 trigger expression of ZEB1 proteins. As a result, the ZEB family controls cancer progression and metastasis.

**Figure 3:**
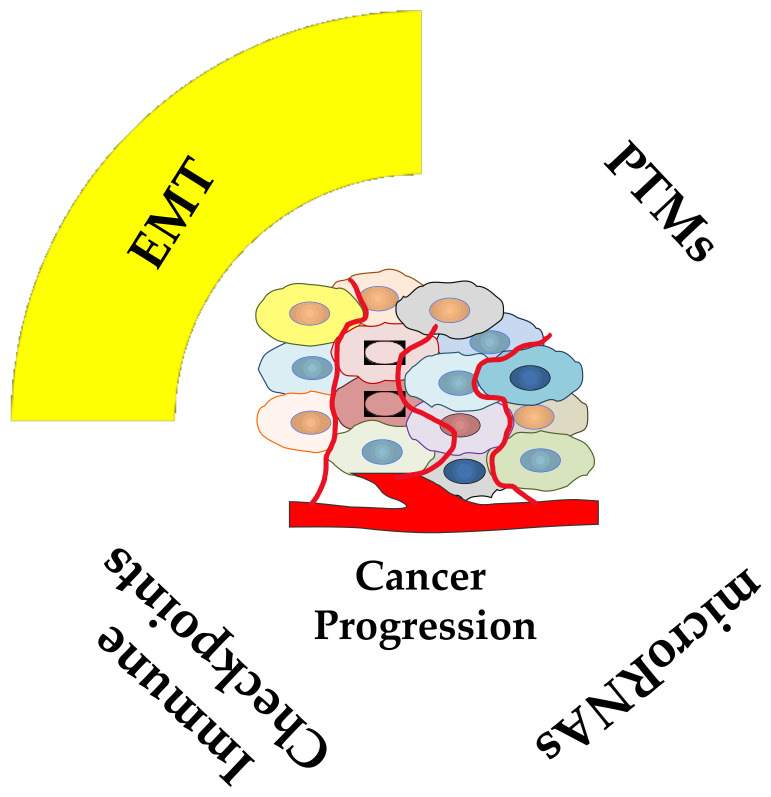
Regulation of ZEB family in cancer progression. PTMs, EMT, miRNA and immune checkpoints of ZEBs functionally are linked to cancer progression.

ZEB protein is involved in tumour invasion and metastasis in the invasive front of carcinomas by EMT induction. ZEB1 is highly expressed in several tumours, including breast [6,7], pancreatic [9,27,34], colorectal [35], gastric [36,37], lung [38,39,40], uterine [41], hepatocellular carcinoma [42], prostate [43,44] and lymphoma [45] cancers. In these tumours, ZEB1 expression correlates with the loss of E-cadherin and is associated with advanced disease or metastasis, indicating the relevance of ZEB1 induction of EMT and tumour progression [13]. Mechanistically, TGF-β enhances pSMAD2/3 and ZEB1 [46] and ZEB2 [47] expression to increase tumour invasion. The β-catenin translocates into the nucleus to activate ZEB1 [48] transcription. WNT signaling induces ZEB2 expression in tumour metastasis [49]. Activation of MEK1/2 and ERK1/2 promotes tumour progression by ZEB1 [50] and ZEB2 [51]. TNF-α induces the mesenchymal phenotype via NF-κB, ZEB1 and ZEB2 signaling [52]. Fos-related antigen 1 (Fra-1) is a member of the Fos family that dimerizes with Jun proteins to form AP-1. Fra-1 induces EMT by modulating ZEB1, ZEB2 and TGFβ expression [53]. E2F1, a transcription factor, regulates EMT and metastasis by increasing ZEB2 expression in small-cell lung cancer [54]. ZEB2 is coexpressed with the POU family and upregulates EMT induction [55]. PRC2-mediated ZEB2 expression represses PTM by SUMOlation [56]. FOXO1, a member of the FOXO family of transcription factors (FoxOs), binds the ZEB2 promoter and destabilizes the ZEB2 mRNA. As a result, it inhibits ZEB2-induced EMT [57] (Figure 2). Loss of E-cadherin is a casual prerequisite for progressing from adenocarcinoma to invasive carcinomas by genetic and epigenetic mechanisms during malignant transformation [8]. In analogy with their function, ZEB1 lose the epithelial phenotype and gain the mesenchymal phenotype with motile and migratory abilities in cancer [5]. Moreover, ZEB1/miR-200 plays an essential role in embryonic development and malignant tumour progression [58]. ZEB1 is an essential factor in the regulation of the initiation and development of tumours through EMT (Figure 3).

In the genetically engineered mouse model (GEMM), *ZEB1* knockout mice die perinatally, exhibiting respiratory failure; severe T cell deficiency of the thymus; and various skeletal defects, including craniofacial abnormalities, limb and sternum defects, and malformed ribs [59]. These developmental defects are associated with mesenchymal-epithelial transition, as evidenced by the re-expression of E-cadherin and loss of vimentin in several tissues and embryonic fibroblasts [60]. In addition, ZEB1 is a crucial factor for local invasion, colonisation capacities and distant metastasis in the Pdx1-Cre-mediated mutant KRAS and the p53 pancreatic cancer mouse (KPC) model [9]. ZEB1 was also shown to affect p53 and RB-dependent oncosuppressive pathways and to prevent senescence and apoptosis, two critical barriers against tumour development. In line with this notion, mouse embryonic fibroblasts (MEF) from *ZEB1* knockout mice undergo early replicative senescence.

ZEB2 is expressed in several tumours, including metastatic ovarian and breast carcinoma [61], pancreatic cancer [62], oral squamous cell carcinomas [63], gastric cancer [64], bladder cancer [65] and glioma [66]. The expressed ZEB2 in tumours is involved in the cell cycle, apoptosis, unregulated cell proliferation, EMT and cancer development and progression [61,64,65,66,67,68]. As T-bet or T-box protein expressed in T cells (TBX21) expression increases, ZEB2 is induced in natural killer (NK) cells. Interaction of ZEB2 and T-bet is required for NK cell maturation to suppress lung melanoma [69]. AKT promotes ZEB2 expression through the nuclear factor-kappa-light-chain-enhancer of activated B cells (NF-κB) pathway in squamous cell carcinoma (SCC) lines [70].

ZEBs also regulate immune checkpoints, evading immune destruction in tumour progression and the microenvironment [71,72]. Programmed death protein 1 (PD-1, encoded by the *PDCD1* gene) and its ligand programmed death-ligand 1 (PD-L1, encoded by the *CD274* gene) function in the immunotherapy of cancer by evading T cell immunity [73]. ZEB1-induced PD-L1 is highly expressed in lung cancer cells [74]. SNHG14/miR-5590-3p/ZEB1 promotes B cell lymphoma progression and evades immunity evasion by exerting PD-1/PD-L1 [75]. ZEB1 is a key target of the melanoma immune escape [76]. ZEB-1 and miR-200 upregulated PD-L1 in breast cancer [77] (Figure 3).

## 4. Post-Translational Modifications of ZEBs in Cancer Progression

PTMs are covalent modifications that occur after the transcript has been translated into proteins, such as the ZEB1, ZEB2, SNAIL (*SNAI1*), SLUG (*SNAI*2) and twist-related (Twist 1) proteins. The human *SNAI1* is located on chromosome 20q13.13 and encodes the 264-amino acid Snail protein. It is a member of the Snail superfamily, and acts as a transcriptional regulator of EMT [78]. The human *SNAI*2 is located on chromosome 8q11.21 and encodes the 268-amino acid Slug protein. Slug binds the nuclear receptor corepressor (NCoR) and C-terminal binding protein 1 (CtBP1) to stabilize Slug and inhibit the expression of E-cadherin [79]. The human *Twist1* genes are located on chromosome 7p21.2 and encodes the 202-amino acid Twist1 protein. The Twist1 plays a critical role in the progression of cancer by modulating EMT [80,81]. These covalent modifications include adding a modifying chemical group or another small protein to one or more residues of the target protein [82]. PTM can occur within the protein on single or multiple residues, undergoing the same or different modifications [83]. Table 1 provides an overview of the molecular mechanisms and biological functions of PTMs of ZEBs in cancer progression.

### 4.1. Phosphorylation

Protein phosphorylation is the most common PTM and is essential for regulating multiple molecular pathways involved in processes such as metabolism, transcription, differentiation and apoptosis. Protein kinases (PK) catalyse phosphorylation by promoting the transfer of ATP’s γ-phosphate to serine, threonine or tyrosine residues on the target proteins. Protein phosphatases (PP) catalyse the reverse process [96,97].

Phosphorylation is a PTM that is known to control ZEB1. Zhang et al. [14] showed that the EMT regulator ZEB1 promotes DNA damage response (DDR) and tumour radioresistance. This regulation is initiated by the phosphorylation and stabilisation of ZEB1 by ATM serine-threonine kinase and is mediated by checkpoint kinase 1 (CHK1) stabilisation by a ZEB1-interacting deubiquitylase, USP7 [14]. Moreover, various CHK1 inhibitors have been tested in anti-cancer clinical trials and warrant investigation as candidate radiosensitising agents for breast tumours with high levels of ZEB1 [98].

Tyrosine kinase receptors related to ZEB1 can also activate EMT. The disassembly of tight junctions during EMT can also be SMAD-independent. TGFβRI and Par6 coexist in tight junctions. On stimulation of TGFβ1, the TβRI-TβRII hetero-dimerise results in a complex containing TβRII/TβRI and Par6 in each tight junction. This interaction results in the phosphorylation of Par6 at Ser345, which is mediated by TβRII [99]. Phosphorylated Par6 interacts with the E3-ubiquitin ligase SMURF-1 that targets RhoA for degradation, leading to the disassembly of tight junctions. Therefore, TGFβ signalling has good therapeutic value [100,101]. GSK3β phosphorylates ZEB2 at Ser705 and Tyr802 in colorectal cancer; this induces EMT, colorectal cancer-cancer stem-like cell properties and metastasis [93].

### 4.2. SUMOylation

SUMOylation is another PTM characterised by the reversible binding of a small ubiquitin-like modifier (SUMO) to the target protein. The three-dimensional structure of SUMO is similar to ubiquitin [102,103]. SUMO modulates DNA damage repair [104,105,106], immune responses, carcinogenesis, cell cycle progression [102,107] and apoptosis. Therefore, SUMOylation is attributed to cancer progression and might function as an actional therapeutic target for cancer [108]. Olig2 SUMOylation has been demonstrated to protect against the genotoxic damage response by antagonising p53 gene targeting [109]. Furthermore, the EMT-related transcription factor ZEB1 was SUMO-modified, and its levels decreased in Senp1-silenced HCC cells [85].

Members of the miR-200 family act as tumour-suppressive miRNAs, enhancing the expression of E-cadherin and suppressing the expression of ZEB1 and ZEB2. The overexpression of miR-200 results in a reduced expression of ZEB transcription factors and enhanced expression of epithelial makers [21,58,110]. In pancreatic cancer, FoxM1 is overexpressed and promotes EMT by the up-regulation of mesenchymal cell markers, such as ZEB1, ZEB2, SLUG and vimentin [111]. Pc2-mediated SUMOylation of ZEB2 (Lys391, Lys866) modulates the repression of E-cadherin transcription [56].

### 4.3. Ubiquitination and Deubiquitination

Ubiquitination regulates the activity and levels of proteins. It is associated with various disease such as cancer, autoimmunity and inflammation [112,113]. Therefore, understanding of ubiquitination processes could provide opportunities for the development of new therapeutics [114]. The seven-in-absentia (Siah) ubiquitin ligases have been reported to decrease the stability of the ZEB1 protein through the ubiquitin-proteasome pathway; this subsequently affects the EMT process in mammalian cancer cells [86]. Deubiquitinating enzymes (DUBs) are key components of the ubiquitin-proteasome system (UPS) that remove ubiquitin chains from their protein substrates [115]. A large body of evidence suggests that the dysfunction of DUBs is responsible for many pathologies, including cancer [116,117]. More recently, there have been reports of associations between DUBs and metastasis in various cancer types. For example, the ectopic expression of ubiquitin-specific protease 14 (USP14) is associated with liver and lymph node metastasis in colorectal cancer [118]. Multiple deubiquitinating enzymes, including USP26, ubiquitin thioesterase (OTUB1) and pMSD3, have been shown to promote the metastasis of oesophageal squamous cell carcinoma through the stabilisation of Snail, which is another EMT transcription factor [119,120,121]. Notably, WP1130 is a partially selective inhibitor of several DUBs, including USP9X, USP5, USP14 and UCH37, and has been shown to trigger a rapid accumulation of polyubiquitinated proteins into aggresomes and induce breast tumour regression [122]; this suggests a potential role for DUBs as therapeutic targets in the treatment of cancer metastasis. Zhang et al. [123] reported that the CDK4/6-USP51-ZEB1 axis plays a vital role in breast cancer metastasis and could be a viable therapeutic target for treating advanced human cancers. In particular, USP51 deubiquitinates ZEB1’s *N*-terminal region and minces metastasis and therapy resistance in breast cancer [87]. USP18 also deubiquitinates ZEB1 and enhances EMT and metastasis in oesophageal squamous cell carcinomas. Furthermore, CSN5 regulates the levels of ZEB1 ubiquitination in renal cell carcinoma and could be a potential therapeutic target [88]. The SBD domain of ZEB2 is required for ubiquitination mediated by Fbxo45 and K48-linkage poly-ubiquitination on ZEB2 [94].

### 4.4. Acetylation and Deacetylation

Acetylation and deacetylation play roles in the regulation of transcriptional activation, nuclear localisation and DNA binding. In addition, they are associated with signalling pathways, cell cycle and ZEB1 at Lys741, LysK774 and Lys775 was acetylated by P/CAF [25]. Tip60, a cell-type-specific transcriptional regulator, acetylates the N-terminal of ZEB1 [90]. ZEB1 recruits the histone deacetylases HDAC1 and HDAC2, which inhibit E-cadherin expression in pancreatic cancer [91].

## 5. Targeting ZEB Modification for Cancer Therapy and Therapeutic Resistance

ZEB is highly expressed in several cancers. Inhibiting the biological function of ZEB is a new approach in cancer therapy. The expression, stability, and localization of ZEB could interfere with different interactions in cancer patients. Phosphorylation of retinoblastoma (Rb) enhances the progression and metastasis of several cancers. The mechanism of RB in cancer is associated with the regulation of cyclins and cyclin-dependent kinase. Rb dephosphorylation regulates EMT by inhibiting the ZEB1 transcriptional activity in breast cancer [124]. There are ZEB PTMs in resistance to various anticancer therapies [125]. The expression level of ZEB1 by induction ATM activation occurs in chemoresistance in human breast cancer [126]. ZEB1 regulates tumor radioresistance by deubiquitylation of USP7. This in turn upregulates homologous recombination-dependent DNA damage repair (DDR) and tumor radioresistance [14]. ZEB1 also modulates the radiotherapy resistance by the inhibition miR-205 [127].

## 6. Summary and Perspective

Highly expressed ZEBs are significantly associated with the tumourigenesis and metastasis of various cancers. Furthermore, the ZEBs, including ZEB1 and ZEB2, are transcription factors that control EMT in tumour progression; this is a process in which tumour cells within the primary tumour lose their cell junctions and their epithelial morphology changes to fibroblastoid morphology. Therefore, understanding the mechanism of ZEBs’ contribution to metastasis is paramount in improving cancer treatment outcomes. However, very little research has been done to target PTMs of ZEBs in cancers. We hypothesised that identifying PTM inhibitors represents an underexplored area of research that may hold significant potential in developing future cancer treatments. Furthermore, identifying ZEBs’ post-transcriptional and PTMs is vital, given that these changes could be identified in the primary tumour before metastasis occurs. Such knowledge can allow clinicians to better predict the patients who have genotypes more likely to follow an aggressive clinical course prone to the development of metastases. These patients could then be treated with different approaches from the onset of the disease to reduce the risk of metastasis, allow for better prognoses and ultimately, enhance survival.

We highlighted the current understanding of the regulation and underlying molecular mechanisms of the ZEB family members’ PTMs in cancer progression; this may provide new insights into developing novel cancer therapeutic strategies and opportunities.

## Figures and Tables

**Table 1:** Functions of ZEBs-PTMs.

PTMs Type	PTM Sites	Kinase/Enzyme	Biological Function	Cancer Type	Ref.
ZEB1					
Phosphorylation	Thr867	ERK	Inhibition of the nuclear localisation of ZEB1	-	[84]
	Thr851, Ser852, Ser853	PKC	Inhibition of the nuclear localisation of ZEB1	-	[84]
	Ser585	ATM	Promotes DDR and tumour radioresistance	BC	[14]
SUMOylation	-	Senp1	Promotes migration and EMT.	HCC	[85]
Ubiquitination	-	Siah	Promotes cell proliferation and invasion	BC	[86]
Deubiquitination	N-terminal	USP51	Promotes cell proliferation and invasion	BC	[87]
		CSN5	Promotes metastasis and EMT	RCC	[88]
		USP18	Promotes EMT	ESCC	[89]
Acetylation	Lys741, Lys774, Lys775	P/CAF	Promotes the formation of a p300-SMAD transcriptional complex		[25]
	N-terminal	TIP60	Corepressor of the ZEB	T lymphoma	[90]
Deacetylation		HDAC1/2	Promotes EMT	PAAD	[91,92]
ZEB2					
Phosphorylation	Ser705, Tyr802	GSK-3β	Promotes metastasis and chemoresistance	CRC	[93]
SUMOylation	Lys391, Lys866	Pc2	Promotes EMT		[56]
Ubiquitination	Lys48	FBXO45	Promotes EMT initiation and cancer progression		[94]
		FBXL14	Promotes EMT	COAD	[95]
		FBXW7	Promotes metastasis and chemoresistance	CRC	[93]

BC, breast cancer; HCC, hepatocellular carcinoma; RCC, renal cell carcinoma; ESCC, oesophageal squamous cell carcinomas; CESC, cervical squamous cell carcinoma and endocervical adenocarcinoma; PAAD, pancreatic adenocarcinoma; CRC, colorectal cancer; COAD, colon adenocarcinoma; ERK, extracellular signal-regulated kinase; PKC, protein kinase C; ATM, ataxia–telangiectasia mutated kinase; USP51, ubiquitin-specific peptidase 51; CSN5, COP9 signalosome subunit 5; USP18, ubiquitin-specific peptidase 18; PCAF, p300/CBP-associated factor; TIP60, tat-interacting protein of 60 kDa; HDAC1/2, histone deacetylase 1/2; GSK3β, glycogen synthase kinase 3 beta; FBXO45, F-box only protein 45; FBXL14, F-Box and leucine-rich repeat protein 14; FBXW7, F-box/WD repeat-containing protein 7.

## Data Availability

Not applicable.

## Abbreviations

ATP	adenosine triphosphate
BMP	bone morphogenetic proteins
CBD	coactivator binding domain
CHK1	checkpoint kinase 1
CID	CtBP interaction domain
CtBP1	C-terminal binding protein 1
DDR	DNA damage response
DUB	deubiquitinating enzymes
EMT	epithelial-mesenchymal transition
ERK	extracellular signal-regulated kinase
FoxOs	FOXO family of transcription factors
Fra-1	Fos-related antigen 1
GEMM	genetically engineered mouse model
HD	homeodomain
KPC	KRAS and the p53 pancreatic cancer mouse
MEF	mouse embryonic fibroblasts
miRNAs	micro-RNAs
NCoR	nuclear receptor corepressor
NF-κB	nuclear factor kappa B
NK	natural killer
NLS	nuclear localization signal
OTUB1	ubiquitin thioesterase
PD-1	programmed death protein 1
PD-L1	programmed death-ligand 1
PK	Protein kinases
PP	Protein phosphatases
PTM	post-translational modification
Siah	seven-in-absentia
SID	SMAD interaction domain
SUMO	small ubiquitin-like modifier
TBX21	T-bet or T-box protein expressed in T cells
TGFβ	transforming growth factor beta
TNF	tumour necrosis factor
UPS	ubiquitin-proteasome system
ZEB	zinc finger E-box binding homeobox
ZF	zinc finger
ZFD	zinc finger domain

## References

[1] Siegel R.L., Miller K.D., Fuchs H.E., Jemal A. (2022). Cancer statistics, 2022. CA Cancer J. Clin..

[2] Hay E. (1995). An Overview of Epithelio-Mesenchymal Transformation. Acta Anat..

[3] Thiery J.P., Sleeman J.P. (2006). Complex networks orchestrate epithelial–mesenchymal transitions. Nat. Rev. Mol. Cell Biol..

[4] Thiery J.P., Acloque H., Huang R.Y.J., Nieto M.A. (2009). Epithelial-Mesenchymal Transitions in Development and Disease. Cell.

[5] Kalluri R., Weinberg R.A. (2009). The basics of epithelial-mesenchymal transition. J. Clin. Investig..

[6] Yu J.-M., Sun W., Hua F., Xie J., Lin H., Zhou D.-D., Hu Z.-W. (2015). BCL6 induces EMT by promoting the ZEB1-mediated transcription repression of E-cadherin in breast cancer cells. Cancer Lett..

[7] Eger A., Aigner K., Sonderegger S., Dampier B., Oehler S., Schreiber M., Berx G., Cano A., Beug H., Foisner R. (2005). DeltaEF1 is a transcriptional repressor of E-cadherin and regulates epithelial plasticity in breast cancer cells. Oncogene.

[8] Schmalhofer O., Brabletz S., Brabletz T. (2009). E-cadherin, β-catenin, and ZEB1 in malignant progression of cancer. Cancer Metastasis Rev..

[9] Krebs A.M., Mitschke J., Lasierra Losada M., Schmalhofer O., Boerries M., Busch H., Boettcher M., Mougiakakos D., Reichardt W., Bronsert P. (2017). The EMT-activator Zeb1 is a key factor for cell plasticity and promotes metastasis in pancreatic cancer. Nat. Cell Biol..

[10] Wu C.-Y., Tsai Y.-P., Wu M.-Z., Teng S.-C., Wu K.-J. (2012). Epigenetic reprogramming and post-transcriptional regulation during the epithelial–mesenchymal transition. Trends Genet..

[11] Gheldof A., Hulpiau P., van Roy F., De Craene B., Berx G. (2012). Evolutionary functional analysis and molecular regulation of the ZEB transcription factors. Cell. Mol. Life Sci..

[12] Wu L., Zhou Z., Han S., Chen J., Liu Z., Zhang X., Yuan W., Ji J., Shu X. (2020). PLAGL2 promotes epithelial–mesenchymal transition and mediates colorectal cancer metastasis via β-catenin-dependent regulation of ZEB1. Br. J. Cancer.

[13] Zhang P., Sun Y., Ma L. (2015). ZEB1: At the crossroads of epithelial-mesenchymal transition, metastasis and therapy resistance. Cell Cycle.

[14] Zhang P., Wei Y., Wang L., Debeb B.G., Yuan Y., Zhang J., Yuan J., Wang M., Chen D., Sun Y. (2014). ATM-mediated stabilization of ZEB1 promotes DNA damage response and radioresistance through CHK1. Nat. Cell Biol..

[15] Wang H., Xiao Z., Zheng J., Wu J., Hu X.-L., Yang X., Shen Q. (2019). ZEB1 Represses Neural Differentiation and Cooperates with CTBP2 to Dynamically Regulate Cell Migration during Neocortex Development. Cell Rep..

[16] Chang R., Zhang P., You J. (2016). Post-translational modifications of EMT transcriptional factors in cancer metastasis. Open Life Sci..

[17] Chen L., Liu S., Tao Y. (2020). Regulating tumor suppressor genes: Post-translational modifications. Signal Transduct. Target. Ther..

[18] Appel M.J., Bertozzi C.R. (2015). Formylglycine, a Post-Translationally Generated Residue with Unique Catalytic Capabilities and Biotechnology Applications. ACS Chem. Biol..

[19] Okeley N.M., van der Donk W.A. (2000). Novel cofactors via post-translational modifications of enzyme active sites. Chem. Biol..

[20] Dominy J.E., Hwang J., Guo S., Hirschberger L.L., Zhang S., Stipanuk M.H. (2008). Synthesis of Amino Acid Cofactor in Cysteine Dioxygenase Is Regulated by Substrate and Represents a Novel Post-translational Regulation of Activity. J. Biol. Chem..

[21] Hill L., Browne G., Tulchinsky E. (2013). ZEB/miR-200 feedback loop: At the crossroads of signal transduction in cancer. Int. J. Cancer.

[22] Williams T.M., Montoya G., Wu Y., Eddy R.L., Byers M.G., Shows T.B. (1992). The TCF8 gene encoding a zinc finger protein (Nil-2-a) resides on human chromosome 10p11.2. Genomics.

[23] Wakamatsu N., Yamada Y., Yamada K., Ono T., Nomura N., Taniguchi H., Kitoh H., Mutoh N., Yamanaka T., Mushiake K. (2001). Mutations in SIP1, encoding Smad interacting protein-1, cause a form of Hirschsprung disease. Nat. Genet..

[24] Postigo A.A. (2003). Opposing functions of ZEB proteins in the regulation of the TGFbeta/BMP signaling pathway. EMBO J..

[25] Postigo A., Depp J.L., Taylor J.J., Kroll K.L. (2003). Regulation of Smad signaling through a differential recruitment of coactivators and corepressors by ZEB proteins. EMBO J..

[26] Peinado H., Olmeda D., Cano A. (2007). Snail, Zeb and bHLH factors in tumour progression: An alliance against the epithelial phenotype?. Nat. Rev. Cancer.

[27] Wellner U., Brabletz T., Keck T. (2010). ZEB1 in Pancreatic Cancer. Cancers.

[28] Chung D.-W.D., Frausto R.F., Ann L.B., Jang M.S., Aldave A.J., Rosales M.A.B., Silva K.C., Duarte D.A., Rossato F.A., de Faria J.B.L. (2014). Functional Impact of *ZEB1* Mutations Associated With Posterior Polymorphous and Fuchs’ Endothelial Corneal Dystrophies. Investig. Opthalmol. Vis. Sci..

[29] Vandewalle C., van Roy F., Berx G. (2009). The role of the ZEB family of transcription factors in development and disease. Cell. Mol. Life Sci..

[30] Scott C.L., Omilusik K.D. (2019). ZEBs: Novel Players in Immune Cell Development and Function. Trends Immunol..

[31] Verschueren K., Remacle J.E., Collart C., Kraft H., Baker B.S., Tylzanowski P., Nelles L., Wuytens G., Su M.-T., Bodmer R. (1999). SIP1, a Novel Zinc Finger/Homeodomain Repressor, Interacts with Smad Proteins and Binds to 5′-CACCT Sequences in Candidate Target Genes. J. Biol. Chem..

[32] Remacle J.E., Kraft H., Lerchner W., Wuytens G., Collart C., Verschueren K., Smith J., Huylebroeck D. (1999). New mode of DNA binding of multi-zinc finger transcription factors: Delta EF1 family members bind with two hands to two target sites. EMBO J..

[33] Kropivšek K., Pickford J., Carter D.A. (2014). Postnatal Dynamics of Zeb2 Expression in Rat Brain: Analysis of Novel 3′ UTR Sequence Reveals a miR-9 Interacting Site. J. Mol. Neurosci..

[34] Bronsert P., Kohler I., Timme S., Kiefer S., Werner M., Schilling O., Vashist Y., Makowiec F., Brabletz T., Hopt U.T. (2014). Prognostic significance of Zinc finger E-box binding homeobox 1 (ZEB1) expression in cancer cells and cancer-associated fibroblasts in pancreatic head cancer. Surgery.

[35] Zhang G.-J., Zhou T., Tian H.-P., Liu Z.-L., Xia S.-S. (2013). High expression of ZEB1 correlates with liver metastasis and poor prognosis in colorectal cancer. Oncol. Lett..

[36] Okugawa Y., Toiyama Y., Tanaka K., Matsusita K., Fujikawa H., Saigusa S., Ohi M., Inoue Y., Mohri Y., Uchida K. (2012). Clinical significance of zinc finger E-box binding homeobox 1 (ZEB1) in human gastric cancer. J. Surg. Oncol..

[37] Jia B., Liu H., Kong Q., Li B. (2012). Overexpression of ZEB1 associated with metastasis and invasion in patients with gastric carcinoma. Mol. Cell. Biochem..

[38] Zhang J., Lu C., Zhang J., Kang J., Cao C., Li M. (2013). Involvement of ZEB1 and E-cadherin in the invasion of lung squamous cell carcinoma. Mol. Biol. Rep..

[39] Matsubara D., Kishaba Y., Yoshimoto T., Sakuma Y., Sakatani T., Tamura T., Endo S., Sugiyama Y., Murakami Y., Niki T. (2014). Immunohistochemical analysis of the expression of E-cadherin and ZEB1 in non-small cell lung cancer. Pathol. Int..

[40] Larsen J., Nathan V., Osborne J.K., Farrow R.K., Deb D., Sullivan J.P., Dospoy P.D., Augustyn A., Hight S.K., Sato M. (2016). ZEB1 drives epithelial-to-mesenchymal transition in lung cancer. J. Clin. Investig..

[41] Spoelstra N.S., Manning N.G., Higashi Y., Darling D., Singh M., Shroyer K.R., Broaddus R.R., Horwitz K.B., Richer J.K. (2006). The Transcription Factor ZEB1 Is Aberrantly Expressed in Aggressive Uterine Cancers. Cancer Res..

[42] Zhou Y.-M., Cao L., Li B., Zhang R.-X., Sui C.-J., Yin Z.-F., Yang J.-M. (2012). Clinicopathological Significance of ZEB1 Protein in Patients with Hepatocellular Carcinoma. Ann. Surg. Oncol..

[43] Contreras H., Orellana-Serradell O., Herrera D., Castellón E. (2019). The transcription factor ZEB1 promotes chemoresistance in prostate cancer cell lines. Asian J. Androl..

[44] Wang H., Huang B., Li B.M., Cao K.Y., Mo C.Q., Jiang S.J., Pan J.C., Wang Z.R., Lin H.Y., Wang D.H. (2018). ZEB 1-mediated vasculogenic mimicry formation associates with epithelial–mesenchymal transition and cancer stem cell phenotypes in prostate cancer. J. Cell. Mol. Med..

[45] Wu C., Li J., Tian C., Shi W., Jiang H., Zhang Z., Wang H., Zhang Q., Sun W., Sun P. (2018). Epigenetic dysregulation of ZEB1 is involved in LMO2-promoted T-cell acute lymphoblastic leukaemia leukaemogenesis. Biochim. Biophys. Acta (BBA)—Mol. Basis Dis..

[46] Joseph J.V., Conroy S., Tomar T., Eggens-Meijer E., Bhat K., Copray S., Walenkamp A.M.E., Boddeke E., Balasubramanyian V., Wagemakers M. (2014). TGF-β is an inducer of ZEB1-dependent mesenchymal transdifferentiation in glioblastoma that is associated with tumor invasion. Cell Death Dis..

[47] Massagué J. (2008). TGFbeta in Cancer. Cell.

[48] Kalra R.S., Chaudhary A., Yoon A.-R., Bhargava P., Omar A., Garg S., Yun C.-O., Kaul S.C., Wadhwa R. (2018). CARF enrichment promotes epithelial–mesenchymal transition via Wnt/β-catenin signaling: Its clinical relevance and potential as a therapeutic target. Oncogenesis.

[49] Xu J., Lamouille S., Derynck R. (2009). TGF-beta-induced epithelial to mesenchymal transition. Cell Res..

[50] Han Y., Luo Y., Wang Y., Chen Y., Li M., Jiang Y. (2016). Hepatocyte growth factor increases the invasive potential of PC-3 human prostate cancer cells via an ERK/MAPK and Zeb-1 signaling pathway. Oncol. Lett..

[51] Sinh N.D., Endo K., Miyazawa K., Saitoh M. (2017). Ets1 and ESE1 reciprocally regulate expression of ZEB1/ZEB2, dependent on ERK1/2 activity, in breast cancer cells. Cancer Sci..

[52] Chua H.L., Bhat-Nakshatri P., Clare S.E., Morimiya A., Badve S., Nakshatri H. (2007). NF-κB represses E-cadherin expression and enhances epithelial to mesenchymal transition of mammary epithelial cells: Potential involvement of ZEB-1 and ZEB-2. Oncogene.

[53] Bakiri L., Macho-Maschler S., Custic I., Niemiec J., Guío-Carrión A., Hasenfuss S.C., Eger A., Müller M., Beug H., Wagner E.F. (2015). Fra-1/AP-1 induces EMT in mammary epithelial cells by modulating Zeb1/2 and TGFβ expression. Cell Death Differ..

[54] Wang T., Chen X., Qiao W., Kong L., Sun D., Li Z. (2017). Transcription factor E2F1 promotes EMT by regulating ZEB2 in small cell lung cancer. BMC Cancer.

[55] Katoh M. (2009). Integrative genomic analyses of ZEB2: Transcriptional regulation of ZEB2 based on SMADs, ETS1, HIF1α, POU/OCT, and NF-κB. Int. J. Oncol..

[56] Long J., Zuo D., Park M. (2005). Pc2-mediated Sumoylation of Smad-interacting Protein 1 Attenuates Transcriptional Repression of E-cadherin. J. Biol. Chem..

[57] Dong T., Zhang Y., Chen Y., Liu P., An T., Zhang J., Yang H., Zhu W., Yang X. (2017). FOXO1 inhibits the invasion and metastasis of hepatocellular carcinoma by reversing ZEB2-induced epithelial-mesenchymal transition. Oncotarget.

[58] Brabletz S., Brabletz T. (2010). The ZEB/miR-200 feedback loop—A motor of cellular plasticity in development and cancer?. EMBO Rep..

[59] Takagi T., Moribe H., Kondoh H., Higashi Y. (1998). DeltaEF1, a zinc finger and homeodomain transcription factor, is required for skeleton patterning in multiple lineages. Development.

[60] Liu Y., El-Naggar S., Darling D.S., Higashi Y., Dean D.C. (2008). Zeb1 links epithelial-mesenchymal transition and cellular senescence. Development.

[61] Elloul S., Elstrand M.B., Nesland J.M., Tropé C.G., Kvalheim G., Goldberg I., Reich R., Davidson B. (2005). Snail, Slug, and Smad-interacting protein 1 as novel parameters of disease aggressiveness in metastatic ovarian and breast carcinoma. Cancer.

[62] Imamichi Y., König A., Gress T., Menke A. (2007). Collagen type I-induced Smad-interacting protein 1 expression downregulates E-cadherin in pancreatic cancer. Oncogene.

[63] Maeda G., Chiba T., Okazaki M., Satoh T., Taya Y., Aoba T., Kato K., Kawashiri S., Imai K. (2005). Expression of SIP1 in oral squamous cell carcinomas: Implications for E-cadherin expression and tumor progression. Int. J. Oncol..

[64] Rosivatz E., Becker I., Specht K., Fricke E., Luber B., Busch R., Höfler H., Becker K.-F. (2002). Differential Expression of the Epithelial-Mesenchymal Transition Regulators Snail, SIP1, and Twist in Gastric Cancer. Am. J. Pathol..

[65] Sayan A.E., Griffiths T.R., Pal R., Browne G.J., Ruddick A., Yagci T., Edwards R., Mayer N.J., Qazi H., Goyal S. (2009). SIP1 protein protects cells from DNA damage-induced apoptosis and has independent prognostic value in bladder cancer. Proc. Natl. Acad. Sci. USA.

[66] Qi S., Song Y., Peng Y., Wang H., Long H., Yu X., Li Z., Fang L., Wu A., Luo W. (2012). ZEB2 Mediates Multiple Pathways Regulating Cell Proliferation, Migration, Invasion, and Apoptosis in Glioma. PLoS ONE.

[67] Mejlvang J., Kriajevska M., Vandewalle C., Chernova T., Sayan A.E., Berx G., Mellon J.K., Tulchinsky E. (2007). Direct Repression of Cyclin D1 by SIP1 Attenuates Cell Cycle Progression in Cells Undergoing an Epithelial Mesenchymal Transition. Mol. Biol. Cell.

[68] Browne G., Sayan A.E., Tulchinsky E. (2010). ZEB proteins link cell motility with cell cycle control and cell survival in cancer. Cell Cycle.

[69] Van Helden M.J., Goossens S., Daussy C., Mathieu A.-L., Faure F., Marçais A., Vandamme N., Farla N., Mayol K., Viel S. (2015). Terminal NK cell maturation is controlled by concerted actions of T-bet and Zeb2 and is essential for melanoma rejection. J. Exp. Med..

[70] Julien S., Puig I., Caretti E., Bonaventure J., Nelles L., van Roy F., Dargemont C., de Herreros A.G., Bellacosa A., Larue L. (2007). Activation of NF-κB by Akt upregulates Snail expression and induces epithelium mesenchyme transition. Oncogene.

[71] Keenan T.E., Burke K.P., Van Allen E.M. (2019). Genomic correlates of response to immune checkpoint blockade. Nat. Med..

[72] Pardoll D.M. (2012). The blockade of immune checkpoints in cancer immunotherapy. Nat. Rev. Cancer.

[73] Cha J.-H., Chan L.-C., Li C.-W., Hsu J.L., Hung M.-C. (2019). Mechanisms Controlling PD-L1 Expression in Cancer. Mol. Cell.

[74] Guo Y., Lu X., Chen Y., Rendon B., Mitchell R.A., Cuatrecasas M., Cortés M., Postigo A., Liu Y., Dean D.C. (2021). Zeb1 induces immune checkpoints to form an immunosuppressive envelope around invading cancer cells. Sci. Adv..

[75] Zhao L., Liu Y., Zhang J., Liu Y., Qi Q. (2019). LncRNA SNHG14/miR-5590-3p/ZEB1 positive feedback loop promoted diffuse large B cell lymphoma progression and immune evasion through regulating PD-1/PD-L1 checkpoint. Cell Death Dis..

[76] Plaschka M., Benboubker V., Grimont M., Berthet J., Tonon L., Lopez J., Le-Bouar M., Balme B., Tondeur G., de la Fouchardière A. (2022). ZEB1 transcription factor promotes immune escape in melanoma. J. Immunother. Cancer.

[77] Noman M.Z., Janji B., Abdou A., Hasmim M., Terry S., Tan T.Z., Mami-Chouaib F., Thiery J.P., Chouaib S. (2017). The immune checkpoint ligand PD-L1 is upregulated in EMT-activated human breast cancer cells by a mechanism involving ZEB-1 and miR-200. OncoImmunology.

[78] Cano A., Pérez-Moreno M.A., Rodrigo I., Locascio A., Blanco M.J., Del Barrio M.G., Portillo F., Nieto M.A. (2000). The transcription factor Snail controls epithelial–mesenchymal transitions by repressing E-cadherin expression. Nat. Cell Biol..

[79] Molina-Ortiz P., Villarejo A., MacPherson M., Santos V., Montes A., Souchelnytskyi S., Portillo F., Cano A. (2012). Characterization of the SNAG and SLUG Domains of Snail2 in the Repression of E-Cadherin and EMT Induction: Modulation by Serine 4 Phosphorylation. PLoS ONE.

[80] Yang J., Mani S.A., Donaher J.L., Ramaswamy S., Itzykson R.A., Come C., Savagner P., Gitelman I., Richardson A., Weinberg R.A. (2004). Twist, a Master Regulator of Morphogenesis, Plays an Essential Role in Tumor Metastasis. Cell.

[81] Chang A.T., Liu Y., Ayyanathan K., Benner C., Jiang Y., Prokop J.W., Paz H., Wang D., Li H.-R., Fu X.-D. (2015). An evolutionarily conserved DNA architecture determines target specificity of the TWIST family bHLH transcription factors. Genes Dev..

[82] Deribe Y.L., Pawson T., Dikic I. (2010). Post-translational modifications in signal integration. Nat. Struct. Mol. Biol..

[83] Pejaver V., Hsu W.-L., Xin F., Dunker A.K., Uversky V.N., Radivojac P. (2014). The structural and functional signatures of proteins that undergo multiple events of post-translational modification. Protein Sci..

[84] Llorens M.C., Lorenzatti G., Cavallo N.L., Vaglienti M.V., Perrone A.P., Carenbauer A.L., Darling D.S., Cabanillas A.M. (2016). Phosphorylation Regulates Functions of ZEB1 Transcription Factor. J. Cell. Physiol..

[85] Zhang W., Sun H., Shi X., Wang H., Cui C., Xiao F., Wu C., Guo X., Wang L. (2016). SENP1 regulates hepatocyte growth factor-induced migration and epithelial-mesenchymal transition of hepatocellular carcinoma. Tumor Biol..

[86] Chen A., Wong C.S., Liu M.C., House C.M., Sceneay J., Bowtell D.D., Thompson E.W., Möller A. (2015). The ubiquitin ligase Siah is a novel regulator of Zeb1 in breast cancer. Oncotarget.

[87] Zhou Z., Zhang P., Hu X., Kim J., Yao F., Xiao Z., Zeng L., Chang L., Sun Y., Ma L. (2017). USP51 promotes deubiquitination and stabilization of ZEB1. Am. J. Cancer Res..

[88] Zhang S., Hong Z., Chai Y., Liu Z., Du Y., Li Q., Liu Q. (2017). CSN5 promotes renal cell carcinoma metastasis and EMT by inhibiting ZEB1 degradation. Biochem. Biophys. Res. Commun..

[89] Song C., Peng J., Wei Y., Shao J., Chen X., Zhang X., Xu J. (2021). USP18 promotes tumor metastasis in esophageal squamous cell carcinomas via deubiquitinating ZEB1. Exp. Cell Res..

[90] Hlubek F., Löhberg C., Meiler J., Jung A., Kirchner T., Brabletz T. (2001). Tip60 Is a Cell-Type-Specific Transcriptional Regulator. J. Biochem..

[91] Aghdassi A., Sendler M., Guenther A., Mayerle J., Behn C.-O., Heidecke C.-D., Friess H., Büchler M., Evert M., Lerch M.M. (2012). Recruitment of histone deacetylases HDAC1 and HDAC2 by the transcriptional repressor ZEB1 downregulates E-cadherin expression in pancreatic cancer. Gut.

[92] Schneider G., Krämer O.H., Saur D. (2012). A ZEB1-HDAC pathway enters the epithelial to mesenchymal transition world in pancreatic cancer. Gut.

[93] Li N., Babaei-Jadidi R., Lorenzi F., Spencer-Dene B., Clarke P., Domingo E., Tulchinsky E., Vries R.G.J., Kerr D., Pan Y. (2019). An FBXW7-ZEB2 axis links EMT and tumour microenvironment to promote colorectal cancer stem cells and chemoresistance. Oncogenesis.

[94] Xu M., Zhu C., Zhao X., Chen C., Zhang H., Yuan H., Deng R., Dou J., Wang Y., Huang J. (2015). Atypical ubiquitin E3 ligase complex Skp1-Pam-Fbxo45 controls the core epithelial-to-mesenchymal transition-inducing transcription factors. Oncotarget.

[95] Lander R., Nordin K., LaBonne C. (2011). The F-box protein Ppa is a common regulator of core EMT factors Twist, Snail, Slug, and Sip1. J. Cell Biol..

[96] Ubersax J.A., Ferrell J.E. (2007). Mechanisms of specificity in protein phosphorylation. Nat. Rev. Mol. Cell Biol..

[97] Johnson L.N. (2009). The regulation of protein phosphorylation. Biochem. Soc. Trans..

[98] Garrett M.D., Collins I. (2011). Anticancer therapy with checkpoint inhibitors: What, where and when?. Trends Pharmacol. Sci..

[99] Ozdamar B., Bose R., Barrios-Rodiles M., Wang H.-R., Zhang Y., Wrana J.L. (2005). Regulation of the Polarity Protein Par6 by TGFß Receptors Controls Epithelial Cell Plasticity. Science.

[100] Neuzillet C., Tijeras-Raballand A., Cohen R., Cros J., Faivre S., Raymond E., de Gramont A. (2015). Targeting the TGFβ pathway for cancer therapy. Pharmacol. Ther..

[101] Zhang B., Halder S.K., Zhang S., Datta P.K. (2009). Targeting transforming growth factor-β signaling in liver metastasis of colon cancer. Cancer Lett..

[102] Bettermann K., Benesch M., Weis S., Haybaeck J. (2012). SUMOylation in carcinogenesis. Cancer Lett..

[103] Bogachek M.V., De Andrade J.P., Weigel R.J. (2015). Regulation of Epithelial–Mesenchymal Transition through SUMOylation of Transcription Factors. Cancer Res..

[104] Sarangi P., Zhao X. (2015). SUMO-mediated regulation of DNA damage repair and responses. Trends Biochem. Sci..

[105] Ulrich H.D. (2012). Ubiquitin and SUMO in DNA repair at a glance. J. Cell Sci..

[106] Jackson S.P., Durocher D. (2013). Regulation of DNA Damage Responses by Ubiquitin and SUMO. Mol. Cell.

[107] Eifler K., Vertegaal A.C. (2015). SUMOylation-Mediated Regulation of Cell Cycle Progression and Cancer. Trends Biochem. Sci..

[108] Han Z.-J., Feng Y.-H., Gu B.-H., Li Y.-M., Chen H. (2018). The post-translational modification, SUMOylation, and cancer (Review). Int. J. Oncol..

[109] Liu H., Weng W., Guo R., Zhou J., Xue J., Zhong S., Cheng J., Zhu M.X., Pan S.-J., Li Y. (2020). Olig2 SUMOylation protects against genotoxic damage response by antagonizing p53 gene targeting. Cell Death Differ..

[110] Wang C.-M., Liu R., Wang L., Nascimento L., Brennan V.C., Yang W.-H. (2014). SUMOylation of FOXM1B Alters Its Transcriptional Activity on Regulation of MiR-200 Family and JNK1 in MCF7 Human Breast Cancer Cells. Int. J. Mol. Sci..

[111] Bao B., Wang Z., Ali S., Kong D., Banerjee S., Ahmad A., Li Y., Azmi A., Miele L., Sarkar F.H. (2011). Over-expression of FoxM1 leads to epithelial-mesenchymal transition and cancer stem cell phenotype in pancreatic cancer cells. J. Cell. Biochem..

[112] Kang J.-B., Shah M.-A., Park D.-J., Koh P.-O. (2022). Retinoic acid regulates the ubiquitin–proteasome system in a middle cerebral artery occlusion animal model. Lab. Anim. Res..

[113] Yi S.S. (2022). Disease predictability review using common biomarkers appearing in diabetic nephropathy and neurodegeneration of experimental animals. Lab. Anim. Res..

[114] Ming-Jer Y., Kai-Cheng H., Tony E.L., Wen-Chang C., Jan-Jong H. (2019). The Role of Ubiquitin-Specific Peptidases in Cancer Progression. J. Biomed. Sci..

[115] Clague M.J., Urbé S., Komander D. (2019). Breaking the chains: Deubiquitylating enzyme specificity begets function. Nat. Rev. Mol. Cell Biol..

[116] Harris I.S., Endress J.E., Coloff J.L., Selfors L.M., McBrayer S.K., Rosenbluth J.M., Takahashi N., Dhakal S., Koduri V., Oser M.G. (2019). Deubiquitinases Maintain Protein Homeostasis and Survival of Cancer Cells upon Glutathione Depletion. Cell Metab..

[117] Zhang Q., Zhang Z.-Y., Du H., Li S.-Z., Tu R., Jia Y.-F., Zheng Z., Song X.-M., Du R.-L., Zhang X.-D. (2019). DUB3 deubiquitinates and stabilizes NRF2 in chemotherapy resistance of colorectal cancer. Cell Death Differ..

[118] Shinji S., Naito Z., Ishiwata S., Ishiwata T., Tanaka N., Furukawa K., Suzuki H., Seya T., Matsuda A., Katsuta M. (2006). Ubiquitin-specific protease 14 expression in colorectal cancer is associated with liver and lymph node metastases. Oncol. Rep..

[119] Li L., Zhou H., Zhu R., Liu Z. (2019). USP26 promotes esophageal squamous cell carcinoma metastasis through stabilizing Snail. Cancer Lett..

[120] Zhou H., Liu Y., Zhu R., Ding F., Cao X., Lin D., Liu Z. (2018). OTUB1 promotes esophageal squamous cell carcinoma metastasis through modulating Snail stability. Oncogene.

[121] Zhu R., Liu Y., Zhou H., Li L., Li Y., Ding F., Cao X., Liu Z. (2018). Deubiquitinating enzyme PSMD14 promotes tumor metastasis through stabilizing SNAIL in human esophageal squamous cell carcinoma. Cancer Lett..

[122] Kapuria V., Peterson L.F., Fang D., Bornmann W.G., Talpaz M., Donato N.J. (2010). Deubiquitinase Inhibition by Small-Molecule WP1130 Triggers Aggresome Formation and Tumor Cell Apoptosis. Cancer Res..

[123] Zhang Z., Li J., Ou Y., Yang G., Deng K., Wang Q., Wang Z., Wang W., Zhang Q., Wang H. (2020). CDK4/6 inhibition blocks cancer metastasis through a USP51-ZEB1-dependent deubiquitination mechanism. Signal Transduct. Target. Ther..

[124] Egger J.V., Lane M.V., Antonucci L.A., Dedi B., Krucher N.A. (2016). Dephosphorylation of the Retinoblastoma protein (Rb) inhibits cancer cell EMT via Zeb. Cancer Biol. Ther..

[125] Caramel J., Ligier M., Puisieux A. (2018). Pleiotropic Roles for ZEB1 in Cancer. Cancer Res.

[126] Zhang X., Zhang Z., Zhang Q., Zhang Q., Sun P., Xiang R., Ren G., Yang S. (2018). ZEB1 confers chemotherapeutic resistance to breast cancer by activating ATM. Cell Death Dis..

[127] Zhang P., Wang L., Rodriguez-Aguayo C., Yuan Y., Debeb B.G., Chen D., Sun Y., You M.J., Liu Y., Dean D.C. (2014). miR-205 acts as a tumour radiosensitizer by targeting ZEB1 and Ubc13. Nat. Commun..

